# Magnesium ions mitigate biofilm formation of *Bacillus* species via downregulation of matrix genes expression

**DOI:** 10.3389/fmicb.2015.00907

**Published:** 2015-09-08

**Authors:** Hilla Oknin, Doron Steinberg, Moshe Shemesh

**Affiliations:** ^1^Department of Food Quality and Safety, Institute for Postharvest Technology and Food Sciences, Agricultural Research Organization, The Volcani CenterBet-Dagan, Israel; ^2^Biofilm Research Laboratory, Faculty of Dental Medicine, Institute of Dental Sciences, Hebrew University-HadassahJerusalem, Israel

**Keywords:** biofilm formation, magnesium ions, food industry, *Bacillus* species, microbial development

## Abstract

The objective of this study was to investigate the effect of Mg^2+^ ions on biofilm formation by *Bacillus* species, which are considered as problematic microorganisms in the food industry. We found that magnesium ions are capable to inhibit significantly biofilm formation of *Bacillus* species at 50 mM concentration and higher. We further report that Mg^2+^ ions don't inhibit bacterial growth at elevated concentrations; hence, the mode of action of Mg^2+^ ions is apparently specific to inhibition of biofilm formation. Biofilm formation depends on the synthesis of extracellular matrix, whose production in *Bacillus subtilis* is specified by two major operons: the *epsA-O* and *tapA* operons. We analyzed the effect of Mg^2+^ ions on matrix gene expression using transcriptional fusions of the promoters for *eps* and *tapA* to the gene encoding β galactosidase. The expression of the two matrix operons was reduced drastically in response to Mg^2+^ ions suggesting about their inhibitory effect on expression of the matrix genes in *B. subtilis*. Since the matrix gene expression is tightly controlled by Spo0A dependent pathway, we conclude that Mg^2+^ ions could affect the signal transduction for biofilm formation through this pathway.

## Introduction

The vast majority of bacteria often grow as elaborate multicellular communities, referred to as biofilms (Hall-Stoodley et al., 2004; Kolter and Greenberg, 2006). Biofilm formation represents one of the most successful strategies for survival in natural environments, which protect bacteria and facilitates growth under unfavorable conditions, such as turbulent flow or limited access to nutrients (Stewart and Costerton, 2001; Hall-Stoodley et al., 2004). Biofilm formation is a multistage process in which cells adhere to a surface through production of an extracellular matrix that is typically composed of polysaccharides, proteins, and nucleic acids (Flemming and Wingender, 2010). These exopolymeric substances often surround and protect the bacteria (Shemesh et al., 2010). Thus, biofilm bacteria are more resistant than planktonic cells to various antimicrobials (Costerton, 1999; Mah and O'Toole, 2001).

Biofilms are problematic in a broad range of areas, and specifically in the food, environmental, and biomedical fields (Simoes et al., 2010). Within food industry, biofilm formation in dairy processing plants is a most significant problem. The major source of the contamination of dairy products is often associated with biofilms (Flint et al., 1997), particularly biofilms formed by members of the *Bacillus* genus (Sharma and Anand, 2002; Simoes et al., 2010). As *Bacillus* species are ubiquitously present in nature, they easily spread through food production systems, and contamination with these species is almost inevitable. The biofilm formed by thermo-resistant *Bacillus* species in a milk line can rapidly grow to such an extent that the passing milk is contaminated with cells released from the biofilm (Wirtanen et al., 1996). Thus, biofilms formed by *Bacillus* species is the major type of hygiene problems in dairy industry.

Clearly, preventing biofilm formation would be a much more desirable option than affecting it in the maturation stage, therefore a range of antimicrobial strategies have been proposed to control biofilms. However, conventional cleaning and disinfection regimens or present antimicrobial strategies may contribute to inefficient biofilm control and to the dissemination of resistance (Simoes et al., 2010). Hence, techniques that are able to prevent or control the formation of unwanted biofilms may have adverse side effects. Therefore, it necessitates looking for other methods to prevent and eradicate bacterial biofilms more successfully.

Environmental factors such as electrolyte concentrations and medium composition may have important impacts on biofilm formation (Song and Leff, 2006; Shemesh et al., 2007). Divalent cations, such as Mg^2+^ and Ca^2+^, can influence biofilm formation directly through their effect on electro-static interactions and indirectly via physiology-dependent attachment processes by acting as important cellular cations and enzyme cofactors (Fletcher, 1988; Malik and Kakii, 2003; Song and Leff, 2006). In spite of the potentially important role, the effect of Mg^2+^ ions on bacterial adhesion and biofilm formation has barely been studied. Mg^2+^ has been shown to influence adherence to surfaces in *Pseudomonas* spp. (Simoni et al., 2000). Moreover, in *Aeromonas hydrophilia*, mutations in Mg^2+^ transport systems result in reduction of swarming and biofilm formation (Merino et al., 2001). Accordingly to Dunne and Burd (1992), 16 mM of Mg^2+^ significantly enhanced *in vitro* adhesion of *Staphylococcus epidermidis* to plastic (Dunne and Burd, 1992). In addition, it was reported that increase in Mg^2+^ concentrations affected positively on biofilm formation by *P. fluorescens* (Song and Leff, 2006). Also, it was hypothesized that low Ca^2+^ or Mg^2+^ concentrations have the potential to inhibit biofilm formation by some *A. flavithermus* and *Geobacillus* spp. strains during the processing of milk formulations (Somerton et al., 2013). Another recent study has showed that biofilm formation decreased with increasing concentration of Mg^2+^ in *Enterobacter cloacae* (Zhou et al., 2014). Although recent studies have shown that magnesium might have diverse effects on biofilms, the effect of Mg^2+^ ions on biofilm formation by sporulation *Bacillus* species remains largely unknown. Therefore, the purpose of this study was to investigate the effect of Mg^2+^ ions on biofilm formation by *Bacillus* species, which are potential importance of biofilm formation in food industrial settings.

## Materials and methods

### Strains and growth media

The *Bacillus subtilis* wild strain NCIB3610 (Branda et al., 2001) and *Bacillus cereus* ATCC 10987 stain, obtained from Michel Gohar's lab collection (INRA, France), were used in this study. For fluorescent microscopy, we used a strain (YC161 with *P*_*spank*_*-gfp*) that produced GFP constitutively (Chai et al., 2011) which was obtained from the laboratory collection of Yunrong Chai (Northeastern University, USA). For routine growth, all strains were propagated in Lysogeny broth (LB; 10 g of tryptone, 5 g of yeast extract and 5 g of NaCl per liter) or on solid LB medium supplemented with 1.5% agar. For biofilm generation, bacteria were grown to stationary phase in LB medium at 37°C in shaking culture to around 1 × 10^8^ CFU per ml. Biofilms were generated at 30°C in the biofilm promoting medium LBGM (LB + 1% (v/v) glycerol + 0.1 mM MnSO_4_) (Shemesh and Chai, 2013). To test the effect of magnesium, sodium or calcium ions on biofilm formation, different concentrations of either MgCl_2_ (Merck KGaA), NaCl (BIO LAB LTD), or CaCl_2_ (Merck KGaA) were added directly into the LBGM medium. For colony type biofilm formation, 3 μl of the cells (around 3 × 10^5^ CFU) was spotted onto LBGM medium solidified with 1.5% agar as described previously (Shemesh and Chai, 2013). Plates were incubated at 30°C for 72 h prior to analysis. For pellicle formation, 5 μl of the cells (around 5 × 10^5^ CFU) was mixed within 4 ml of LBGM broth in 12-well plates (Costar). Plates were incubated at 30°C for 24 h. Images were taken using a Zeiss Stemi 2000-C microscope with an axiocam ERc 5s camera.

For experiments performed with *B. cereus*, bacteria were grown to stationary phase in LB medium at 37°C in shaking culture to around 5 × 10^7^ CFU per ml. For pellicle formation, 5 μl of the cells (around 2.5 × 10^5^ CFU) was mixed within 4 ml of LBGM broth in glass tubes in the presence or absence of different concentration of MgCl_2_. The glass tubes were incubated at 30°C for 24 h.

### Assay of β-galactosidase activity

To analyze the effect of magnesium ions on matrix gene expression we used transcriptional fusions of the promoters for *eps* and *tapA* to the gene encoding β galactosidase (Chai et al., 2008). Samples of generated pellicles as described above were collected and resuspended in phosphate-buffered saline (PBS) buffer. Typical long bundled chains of cells in the biofilm colony were disrupted using mild sonication as described previously (Branda et al., 2006). Optical density of the cell samples was normalized using OD_600_. One milliliter of cell suspensions was collected and assayed for β-Galactosidase activity as described previously (Chai et al., 2008).

### Growth curve analysis

Initially, the cells were grown in shaking cultures over night at 23°C/150 rpm in LB to around 2 × 10^9^ CFU per ml. On the next morning, the cultures were diluted 1:100 (to around 2 × 10^7^ CFU) into LBGM with or without addition of different concentration of MgCl_2_ and incubated at 37°C at 150 rpm. The absorbance of the cultures at 600 nm was measured periodically for each culture for 9 h. Each condition had three replicates, and the growth curve experiments were repeated twice. Representative results are shown.

### Fluorescent microscopy analysis

For fluorescent microscopy, we used a strain YC161 that produced GFP constitutively. The strain was first grown in shaking culture for 5 h 37°C/150 rpm in LB to around 1 × 10^8^ CFU per ml. Next, 5 μl (around 5 × 10^5^ CFU) of suspension from the generated culture was introduced into 4 ml of LBGM medium and incubated at 30°C for 24 h statically. Afterwards, one milliliter of suspension from each sample was collected, mildly sonicated (10 s/20% Amp/5) and centrifuged at 5000 rpm for 2 min. Next, the supernatant was removed and the pellet was resuspended by pipetation. For microscopic observation, 3 μl from the samples were transferred onto a glass slide and visualized in a transmitted light microscope using Nomarski differential interference contrast (DIC), at x40 magnification. A confocal laser scanning microscope was used to visualize GFP expression of strain YC161 using an Olympus IX81 confocal laser scanning microscope (CLSM) (Japan) equipped with 488 nm argon-ion and 543 nm helium neon lasers. For experiments performed with *B. cereus*, the cells were stained with CYTO 9 from the FilmTracer™ LIVE/DEAD Biofilm Viability Kit (Molecular Probes, OR) following instructions of the manufacturer. Fluorescence emission of the stained samples was determined using an Olympus IX81 confocal laser scanning microscope (Japan) equipped with 488 nm argon-ion and 543 nm helium neon lasers.

### Statistical analysis

Statistical analysis was performed using *T*-test to compare the control and tested samples. Statistical significant was determined at *P* < 0.05.

## Results

**Figure 1:**
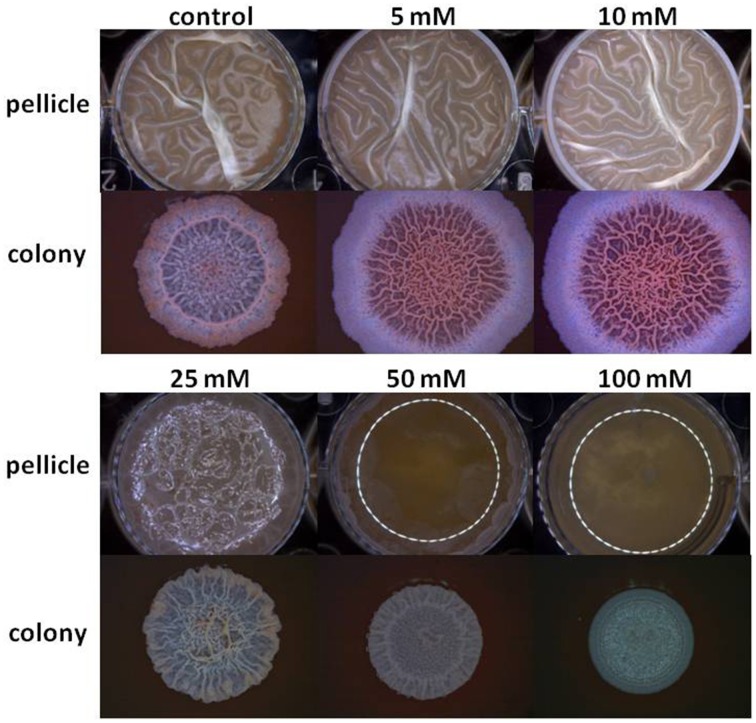
**Mg^2+^ ions block biofilm formation of ***B. subtilis*****. The effect of addition of different concentrations of MgCl_2_ to LBGM medium on pellicle and colony biofilm formation by *B. subtilis* NCIB3610.

The starting point of this investigation was the observation that at the elevated concentrations Mg^2+^ ions could inhibit biofilm formation by *B. subtilis*. As seen in Figure 1, Mg^2+^ ions inhibited notably pellicle formation by *B. subtilis* in a concentration dependent manner. The inhibitory effect of Mg^2+^ ions was not restricted to MgCl_2_ compound since we found that other magnesium salts, such as MgSO_4_ have also inhibited the pellicle formation (data not shown). This indicates that the inhibitory effect of magnesium salts is attributed to Mg^2+^ ions. Moreover, colony type biofilm formation was also inhibited significantly in the presence of high concentrations of Mg^2+^ ions (Figure 1).

To confirm that the significant inhibition in biofilm formation by Mg^2+^ ions is not a result of toxicity to bacterial cells, we tested the effect of different concentrations of Mg^2+^ ions on bacterial growth. As shown in Supplementary Figure 1, the growth curve analysis suggests about very little effect of the Mg^2+^ ions on bacterial growth at the tested concentrations; hence, the mode of action of Mg^2+^ ions is apparently specific to inhibition of biofilm formation.

**Figure 2:**
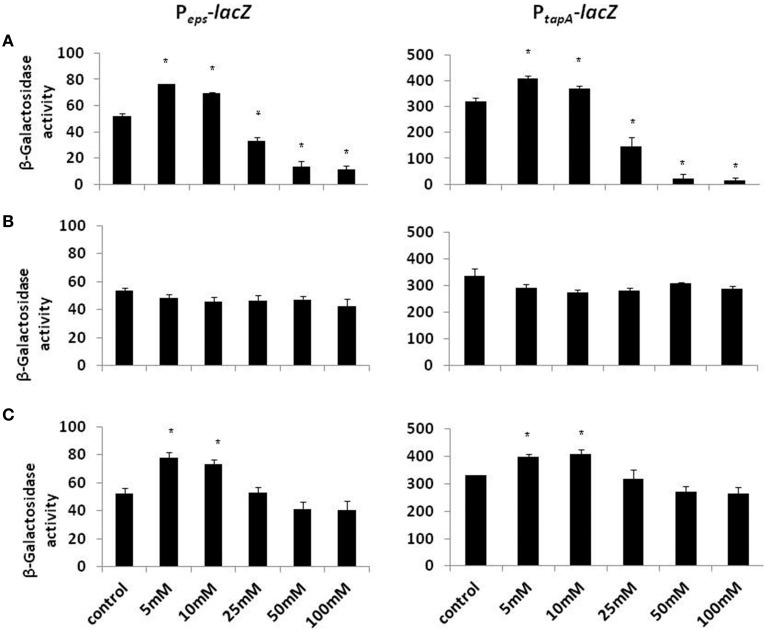
**The effect of Mg^2+^, Ca^2+^, and Na^+^ ions on transcription of the operons responsible for the matrix production**. Transcription of the operons responsible for the matrix production is differentially regulated in response to **(A)** Mg^2+^, **(B)** Na^+^, and **(C)** Ca^2+^ ions. The left panel shows results from RL4548 cells that bear the P_*eps*_-*lacZ* transcriptional fusion and the right panel demonstrates results from RL4582 cells that bear the P_*tapA*_-*lacZ* transcriptional fusion;. ^*^P-value < 0.05 compared to control.

Biofilm formation depends on the synthesis of extracellular matrix, whose production in *B. subtilis* is specified by two major operons: the *epsA-O* and *tapA* operons (Kearns et al., 2005; Branda et al., 2006; Chu et al., 2006). The *epsA-O* operon is responsible for the production of the exopolysaccharides whereas the *tapA* operon is responsible for the production of amyloid-like fibers (Chai et al., 2008; Romero et al., 2010). We hypothesized that the inhibitory effect of Mg^2+^ ions on biofilm formation could be due to down-regulation of the genes involved in matrix synthesis. To test this hypothesis, we analyzed the effect of Mg^2+^ ions on matrix gene expression using transcriptional fusions of the promoters for *epsA-O* and *tapA* to the gene encoding β galactosidase. The expression of the matrix operons was notably reduced in response to the addition of Mg^2+^ ions (Figure 2A). The reduction in *eps* expression was relatively small (around four-fold) but significant, while *tapA* expression was decreased almost 14.5-fold at elevated concentrations of Mg^2+^ ions (Figure 2A). This result suggests that addition of Mg^2+^ ions down regulates expression of the extracellular matrix genes in *B. subtilis*.

**Figure 3:**
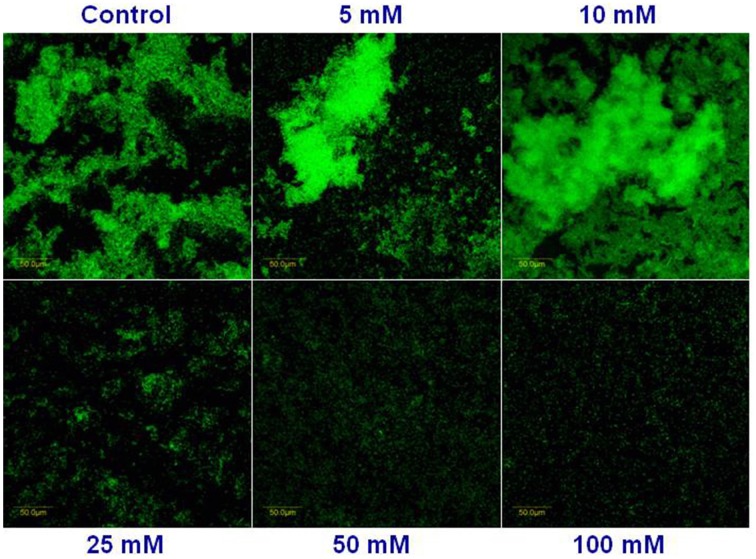
**Mg^2+^ ions block the biofilm bundles formation of ***B. subtilis*****. CLSM images of fluorescently tagged *B. subtilis* cells (YC161 with *P*_*spank*_*-gfp*) following 24 h incubation in biofilm promoting medium.

Next, we visualized microscopically the effect of magnesium ions by testing bundling phenotype of fluorescently tagged *B. subtilis* cells (YC161 with *P*_*spank*_*-gfp*), which produce GFP constitutively (Chai et al., 2011). As seen in Figure 3, there is significant reduction in bundling ability of *B. subtilis* cells in the presence of 25 mM MgCl_2_ and higher. This result further confirms the potential of Mg^2+^ ions to inhibit biofilm formation by *B. subtilis*.

**Figure 4:**
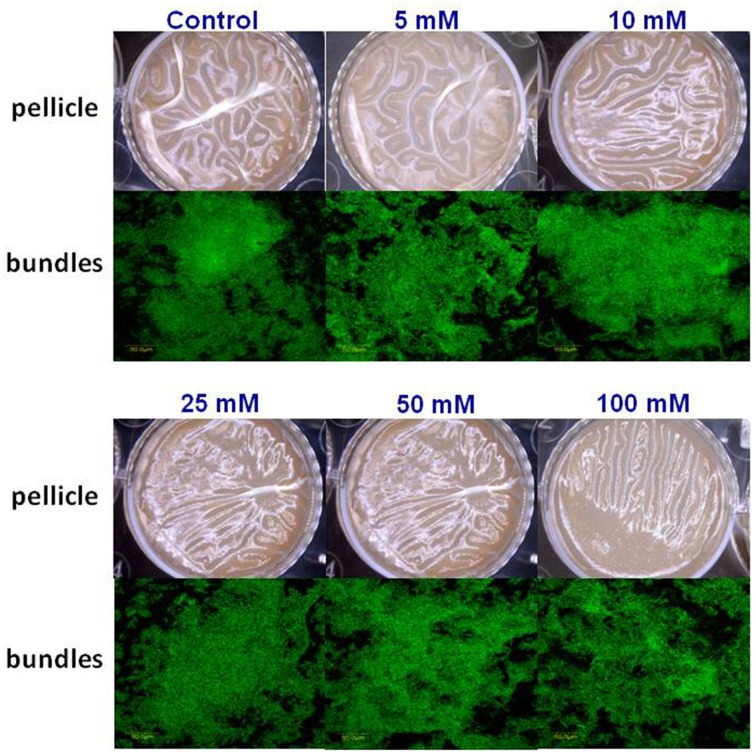
**The effect of Na^+^ ions on biofilm formation by ***B. subtilis*****. The effect of addition of different concentrations of NaCl to LBGM medium on pellicle formation by *B. subtilis* NCIB3610.

**Figure 5:**
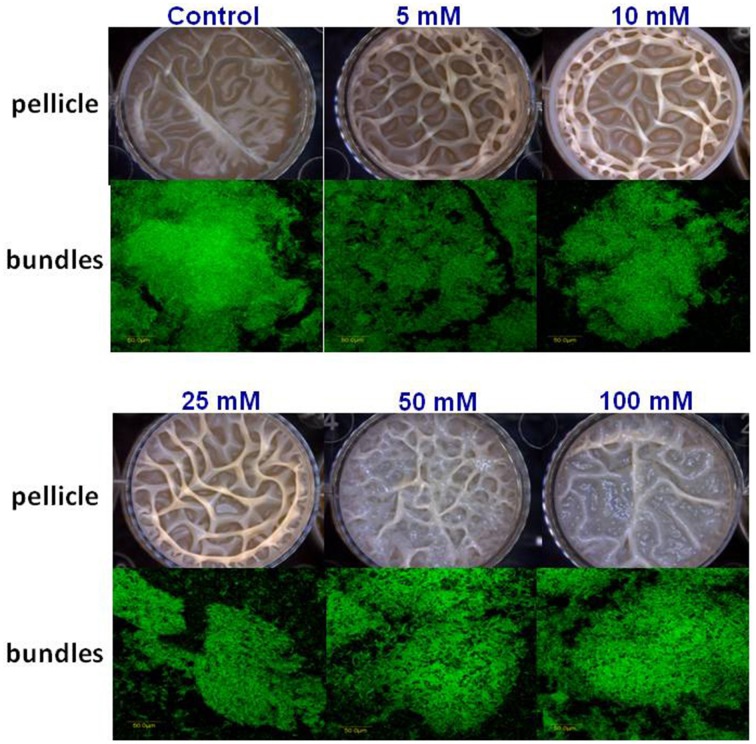
**The effect of Ca^2+^ ions on biofilm formation by ***B. subtilis*****. The effect of addition of different concentrations of CaCl_2_ to LBGM medium on pellicle formation by *B. subtilis* NCIB3610.

Subsequently, we wondered whether other common salts may also affect the biofilm formation at the same concentration as it does MgCl_2_. Therefore, we tested the effect of NaCl and CaCl_2_ on biofilm formation by *B. subtilis*. Notably, none of those compounds could inhibit the biofilm formation in the same manner as MgCl_2_. Although, there was not a significant difference in pellicle formation or bundling ability of *B. subtilis* cells in the presence of NaCl at tested concentrations (Figure 4), nonetheless, NaCl could slightly affect the expression of *eps* operon at 100 mM concentration (Figure 2B). Interestingly, we detected a very slight inhibition in the pellicle formation in the presence of 50 mM or higher concentrations of CaCl_2_ (Figure 5), while expression of the *eps* and *tapA* operons was found to be somewhat downregulated (Figure 2C). It should be noted that neither NaCl nor CaCl_2_ affected notably bacterial growth (Supplementary Figures 2 and 3).

## Discussion

It becomes increasingly clear that most of the bacteria in their natural state exist as surface associated matrix enclosed biofilms. Bacteria are much protected from environmental insults as well as various antimicrobial treatments in the biofilm mode of growth. Our results show that Mg^2+^ inhibits biofilm formation by *B. subtilis* at 25 mM and higher concentration, although at low concentrations (5 and 10 mM) Mg^2+^ enhanced biofilm formation of *B. subtilis*. Apparently, the inhibitory effect of ions is conserved in other *Bacillus* species too. Using CLSM method, we observed a notable inhibition in biofilm formation by *B. cereus* (Supplementary Figure 5), while bacterial growth was not affected in the presence of Mg^2+^ ions (Supplementary Figure 4). Interestingly, the results of our study are in consistence with some of the previous findings regarding the effect of magnesium ions on bacterial adhesion and biofilm formation by different species. Previous studies have shown that Mg^2+^ has varying effects on bacterial adhesion (Marcus et al., 1989; Dunne and Burd, 1992; Tamura et al., 1994), which could be explained due to the difference in bacterial species and Mg^2+^ concentrations used in the various studies. For instance, Tamura et al. (1994) showed that 2 mM magnesium had no significant effect on adherence of *Streptococci*, while higher concentrations enhanced adherence to a small degree (Tamura et al., 1994). Another study showed that at 16 mM magnesium significantly enhanced the *in vitro* adhesion of *S. epidermidis* to plastic surface (Dunne and Burd, 1992). Additional study has showed that Mg^2+^ enhanced adherence of mucoid in one *P. aeruginosa* strain tested and showed no effect on the other (Marcus et al., 1989). It was further found that increase in Mg^2+^ concentrations positively influenced bacterial attachment but the effect changed over time during biofilm formation (Song and Leff, 2006).

It was proposed previously that high Mg^2+^ concentration might contribute to an increase in exopolysaccharide (EPS) production and biofilm stabilization (Costerton et al., 1995). However, it was also found that biofilm formation decreased with increasing concentration of Mg^2+^ in *E. cloacae* (Zhou et al., 2014). In our study, we showed that the expression of the two major operons responsible for biofilm matrix production were reduced notably in response to Mg^2+^ ions, suggesting about an inhibitory effect on expression of the matrix genes in *B. subtilis*. We demonstrated that Mg^2+^ ions are capable to profoundly inhibit biofilm formation of *B. subtilis* at 25 mM concentration and higher. Since the matrix gene expression is tightly controlled by Spo0A~P dependent pathway (Shemesh and Chai, 2013), it is conceivable that Mg^2+^ ions could affect the signal transduction for biofilm formation through this pathway. Although, it is also possible that matrix gene expression is alternatively turned on by a Spo0A~P independent pathway such as YwcC-SlrA pathway (Chai et al., 2009). It will be interesting to further investigate in future studies how the inhibitory effect of Mg^2+^ ions affect a certain signaling pathway involved in biofilm formation.

In our study we decided to determine whether other divalent metal ions such as Ca^2+^ (CaCl_2_) or monovalent metal ions such as Na^+^ (NaCl) can inhibit biofilm formation. Our results showed that Ca^2+^ and Na^+^ ions did not significantly decrease biofilm formation by *B. subtilis*. It is known that the Ca^2+^ ions have beneficial effect of on the mechanical stability of various biofilms (Rose, 2000). Moreover, it is also established that Ca^2+^ ions is important for bacterial biofilm formation (Geesey et al., 2000). Calcium is thought to promote thicker bacterial biofilms, primarily through ionic bridging of the extracellular matrix material (Rose and Turner, 1998; Körstgens et al., 2001). Previous studies have shown that the addition of Ca^2+^ caused a significant increase in *S. paucimobilis* biofilm formation at different concentration levels (Guvensen et al., 2012). Another study has shown that *Pseudoalteromonas spp*. produces larger amounts of biofilm-associated polysaccharide with increased Ca^2+^ (Patrauchan et al., 2005). Additional study showed that the amount of extracellular polysaccharide material of an alginate-producing of *Pseudomonas aeruginosa is* induced as much as eight-fold in response to Ca^2+^ ions (Sarkisova et al., 2005). It was also recently shown that Na^+^ ions could also affect auto-aggregation and biofilm formation in some foodborne pathogens (Xu et al., 2010).

In overall, results of the present study show the inhibitory effect of Mg^2+^ ions on *B. subtilis* biofilm formation as well as reduction in expression of main genes involved in biofilm formation. Findings of this study can open opportunities for development of novel strategies to control biofilm formation in various settings by using small natural molecules. Hence, different magnesium salts can be used to prevent or inhibit bacterial colonization and biofilm formation of *Bacillus* species in industrial and clinical settings.

### Conflict of interest statement

The authors declare that the research was conducted in the absence of any commercial or financial relationships that could be construed as a potential conflict of interest.
