# Supplementary material for: Magnesium ions mitigate biofilm formation of Bacillus species via downregulation of matrix genes expression
Source: Front Microbiol. 2015 Sep 8;6:907. doi: 10.3389/fmicb.2015.00907 (PMC4561805; doi:10.3389/fmicb.2015.00907)
Supplement: Supplementary file 1 [file DataSheet1.DOCX]

**Supplementary Material**

**Magnesium ions mitigate biofilm formation of *Bacillus* species via downregulation of matrix genes expression**

Hilla Oknin^1, 2^, Doron Steinberg^2^, Moshe Shemesh^1*^

^1^Department of Food Quality and Safety, Institute for Postharvest Technology and Food Sciences, Agricultural Research Organization (ARO) The Volcani Center.

^2^Biofilm Research Laboratory, Institute of Dental Sciences, Faculty of Dental Medicine, Hebrew University-Hadassah.

# ^*^Correspondence:

Moshe Shemesh (Ph.D.), Department of Food Quality and Safety, Institute for Postharvest Technology and Food Sciences, Agricultural Research Organization (ARO) The Volcani Center.

**Supplementary Figure 1. The effect of Mg^2+^ ions on bacterial growth.** Growth curves were analyzed in shaking cultures of *B. subtilis* NCIB3610 in LBGM medium at 37°C in the presence of different concentrations of MgCl_2_.

**Supplementary Figure 2. The effect of NaCl on bacterial growth.** Growth curves were analyzed in shaking cultures of *B. subtilis* NCIB3610 in LBGM medium at 37°C in the presence of different concentrations of NaCl.

**Supplementary Figure 3. The effect of CaCl_2_ on bacterial growth.** Growth curves were analyzed in shaking cultures of *B. subtilis* NCIB3610 in LBGM medium at 37°C in the presence of different concentrations of CaCl_2_.

**Supplementary Figure 4.** **The effect of Mg^2+^ ions on bacterial growth of *B. cereus*.** Growth curves were analyzed in shaking cultures of *B. cereus* ATCC 10987 in LBGM medium at 37°C in the presence of different concentrations of MgCl_2_.

**Supplementary Figure 5.** **Mg^2+^ ions block the biofilm bundles formation of *B. cereus*.** CSLM images of *B. cereus* ATCC 10987 cells were stained with CYTO 9 following 24h incubation in biofilm promoting medium.
